# Advance care planning – a multi-centre cluster randomised clinical trial: the research protocol of the ACTION study

**DOI:** 10.1186/s12885-016-2298-x

**Published:** 2016-04-08

**Authors:** Judith A. C. Rietjens, Ida J. Korfage, Lesley Dunleavy, Nancy J. Preston, Lea J. Jabbarian, Caroline Arnfeldt Christensen, Maja de Brito, Francesco Bulli, Glenys Caswell, Branka Červ, Johannes van Delden, Luc Deliens, Giuseppe Gorini, Mogens Groenvold, Dirk Houttekier, Francesca Ingravallo, Marijke C. Kars, Urška Lunder, Guido Miccinesi, Alenka Mimić, Eugenio Paci, Sheila Payne, Suzanne Polinder, Kristian Pollock, Jane Seymour, Anja Simonič, Anna Thit Johnsen, Mariëtte N. Verkissen, Esther de Vries, Andrew Wilcock, Marieke Zwakman, Agnes van der Heide (Pl)

**Affiliations:** Department of Public Health, Erasmus University Medical Center, Rotterdam, The Netherlands; International Observatory on the End-of-Life Care, Lancaster University, Lancaster, LA1 4YG UK; Department of Public Health, University of Copenhagen, Øster Farimagsgade 5, 1014 København, Denmark; The Research Unit, Department of Palliative Medicine, Bispebjerg / Frederiksberg Hospital, Bispebjerg Bakke 23, 2400 København, NV Denmark; Department of Psychology, University of Southern Denmark, Campusvej 55, Odense, 5230 Denmark; Clinical and Descriptive Epidemiology Unit, ISPO Cancer Prevention and Research Institute, Florence, Italy; School of Health Sciences, Sue Ryder Centre for the Study of Supportive, Palliative and End of Life Care, University of Nottingham, Queen’s Medical Centre, Nottingham, UK; University Clinic for Respiratory and Allergic Diseases Golnik, Golnik, Slovenia; Julius Center for Health Sciences and Primary Care, Medical School of Utrecht University, Utrecht, The Netherlands; End-of-life Care Research Group, Vrije Universiteit Brussel (VUB) & Ghent University, Brussels, Belgium; Department of Medical and Surgical Sciences, University of Bologna, Bologna, Italy; Department of Medical Oncology, Ghent University Hospital, Ghent, Belgium; School of Medicine, University of Nottingham, Nottingham University Hospitals NHS Trust, Nottingham, UK

**Keywords:** Advance care planning, Oncology, Quality of life, Medical decision-making

## Abstract

**Background:**

Awareness of preferences regarding medical care should be a central component of the care of patients with advanced cancer. Open communication can facilitate this but can occur in an ad hoc or variable manner. Advance care planning (ACP) is a formalized process of communication between patients, relatives and professional caregivers about patients’ values and care preferences. It raises awareness of the need to anticipate possible future deterioration of health. ACP has the potential to improve current and future healthcare decision-making, provide patients with a sense of control, and improve their quality of life.

**Methods/Design:**

We will study the effects of the ACP program Respecting Choices on the quality of life of patients with advanced lung or colorectal cancer. In a phase III multicenter cluster randomised controlled trial, 22 hospitals in 6 countries will be randomised. In the intervention sites, patients will be offered interviews with a trained facilitator. In the control sites, patients will receive care as usual. In total, 1360 patients will be included. All participating patients will be asked to complete questionnaires at inclusion, and again after 2.5 and 4.5 months. If a patient dies within a year after inclusion, a relative will be asked to complete a questionnaire on end-of-life care. Use of medical care will be assessed by checking medical files. The primary endpoint is patients’ quality of life at 2.5 months post-inclusion. Secondary endpoints are the extent to which care as received is aligned with patients’ preferences, patients’ evaluation of decision-making processes, quality of end-of-life care and cost-effectiveness of the intervention. A complementary qualitative study will be carried out to explore the lived experience of engagement with the Respecting Choices program from the perspectives of patients, their Personal Representatives, healthcare providers and facilitators.

**Discussion:**

Transferring the concept of ACP from care of the elderly to patients with advanced cancer, who on average are younger and retain their mental capacity for a larger part of their disease trajectory, is an important next step in an era of increased focus on patient centered healthcare and shared decision-making.

**Trial registration:**

International Standard Randomised Controlled Trial Number: ISRCTN63110516. Date of registration: 10/3/2014.

## Background

Despite progress in diagnosis and treatment, cancer remains a major life limiting disease, with 14.1 million new cases and 8.2 million deaths worldwide in 2012 [1]. Patients with advanced cancer typically suffer from a reduced quality of life and multiple symptoms, such as pain, fatigue, and dyspnoea, due to their illness and/or its treatment [2]. A diagnosis of advanced cancer often has a tremendous impact on patients’ emotional wellbeing and may result in depression, anxiety and a feeling of loss of control [3, 4]. Ideally, these patients receive patient-centered care, addressing their needs concerning symptom control, psychosocial support, spiritual support, and practical issues. Patients’ preferences regarding care and their wishes concerning their place of residence at the end of life should be central in the decision-making. Currently, treatment aimed at prolonging life has been found to often prevail over care aimed at relieving patients’ suffering and enhancing their quality of life, which may not always be in accordance with patients’ needs and preferences [5].

Timely and efficient communication is an important prerequisite for care that adequately addresses patients’ needs and preferences [6]. However, research findings consistently demonstrate that communication between physicians, patients with advanced cancer and their relatives is complex. Physicians tend to focus on treatment [7], patients may be overwhelmed and unaware of the possibility to opt for treatment aimed at relieving suffering, and relatives may feel stressed and uncertain to be involved in medical decisions without being aware of their beloved one’s preferences [8].

Advance care planning has moved from being a process which aims to elicit specific instructions about medical treatment at the end of life, to being recognized as an opportunity to help patients and their families to prepare, in their own terms, for the changes wrought by serious progressive illness and work with them to plan nursing, social and medical care so that it better fits their needs, hopes and aspirations [9]. ACP is a formalized process of communication between patients, relatives and professional caregivers. It has been defined as “a voluntary process of discussion about future care between an individual and their care providers, irrespective of discipline. […] It is recommended that with the individual’s agreement this discussion is documented, regularly reviewed, and communicated to key persons involved in their care” [10]. ACP promotes discussion of preferences and communication of these preferences to family, friends and healthcare professionals. Patients are encouraged to document their preferences in an advance directive and to review these preferences as circumstances change. Patients are also encouraged to appoint a personal representative, who can express their preferences if they are unable to do so themselves. However, the legal status of advance directives and personal representatives differs across countries. A review of the literature [11] shows that ACP programs have the potential to improve communication between patients and healthcare professionals, increase the quality of life and well-being of patients and their relatives, reduce the use of futile treatments and unnecessary hospitalisations, enhance provision of care that is consistent with patient goals, and increase patients’ satisfaction with care. Other studies have shown that ACP can reduce healthcare costs [12–14]. The Respecting Choices program is one of the most promising ACP program. This program was developed in the US and successfully trialed in a geriatric setting in Australia, showing that patients’ end of life care wishes were much more likely to be known and followed in the intervention group (86 %) compared to the control group (30 %).

Most ACP studies have been performed in the US, amongst nursing home patients with the main aim of establishing patients’ preferences before they lose their competence. We will conduct our study in a European context and hypothesize that ACP can also be effective in improving the quality of life of patients with cancer who often remain competent until death or very close to death. ACP may support them in timely recognizing and continuously expressing their core values and preferences, and to communicate these with their loved ones and professional care givers, which will enable strategic and effective planning of care and decision-making. As a result, care may more adequately address patients’ values and preferences, which may result in improved quality of life and more adequate symptom control, while patients feel more in control and receive less unwanted or futile interventions.

The overall hypothesis that will be studied in the ACTION project is that a formalized ACP program such as Respecting Choices significantly improves the quality of life and reduces the symptom burden of patients with advanced lung or colorectal cancer.

The primary objective is to assess the effect of the Respecting Choices ACP program on the quality of life and symptoms of patients with advanced lung or colorectal cancer.

The secondary objectives are:

1. To assess the effect of the Respecting Choices ACP program on the quality of life and symptoms of patients with advanced cancer in different subgroups (gender, age, education, ethnicity, country and type of cancer).
2. To assess the effect of the Respecting Choices ACP program on the extent to which care as received is in line with patients’ documented preferences, on patients’ evaluation of the quality of the decision-making process, and on how they cope with their illness.
3. To assess patient satisfaction with the Respecting Choices ACP program.
4. To assess the effect of the Respecting Choices ACP program on the quality of end of life care of patients with advanced cancer from the bereaved carers’ perspective, and on the wellbeing of these carers.
5. To assess the cost effectiveness of the Respecting Choices ACP program.
6. To gain insight into how patients, patients’ relatives and professional caregivers experience and respond to facilitated ACP.

## Methods/design

### Study design and setting

We will perform a multicenter cluster-randomised clinical trial in 22 hospitals in six European countries (Belgium, Denmark, Italy, the Netherlands, Slovenia and the United Kingdom). Per country pairs of comparable hospitals (academic/non-academic) will be randomised to provide either ‘care as usual’ supplemented with ACP or ‘care as usual’. Cluster-randomisation prevents healthcare providers from giving patients in the control group (‘care as usual’) more opportunity to discuss their preferences than usual due to their experience with providing the intervention in the intervention group (‘care as usual’ supplemented with ACP). The nature of the intervention makes blinding, for both healthcare professionals and patients and their relatives, impossible.

### Study population

In total, 1,360 patients with advanced lung (*N* = 680) or colorectal cancer (*N* = 680) will be included. Lung and colorectal cancer patients are selected for this study because both types of cancer have high incidence and mortality rates in Europe and affect both sexes; see Table 1 for in- and exclusion criteria. At inclusion, the average life expectancy of these patients is about one year; their minimum estimated life expectancy to be eligible for the study is three months.

**Table 1:** Inclusion and exclusion criteria

Inclusion criteria	Exclusion criteria
Histologically confirmed diagnosis of: • Lung cancer:	Age < 18 years
- small cell - extensive disease/Stage III or IV* - non-small cell - stage III or IV*	Unable to provide consent
• Colorectal cancer: Stage IV or metachronous metastases*	Unable to complete questionnaire in country’s language
*according to 7th edition of TNM classification and staging system	Less than 3 months anticipated life expectancy
Written informed consent to participate	Taking part in a research study that is evaluating palliative care services or communication strategies.
WHO performance status of 0–3.	

### Intervention

In this study, we will evaluate the ACP Respecting Choices program. It involves trained healthcare professionals (“facilitators”, mostly nurses) who assist patients and their relatives in reflecting on the patient’s goals, values and beliefs and in discussing their healthcare wishes [12, 15]. The program also supports people to identify specific activities and experiences that may contribute to, or detract from, their quality of life. Patients are encouraged to appoint a patient representative who preferably also attends the Respecting Choices sessions, and to document their preferences for (future) medical treatment and care in an advance directive; the so-called My Preferences form. These wishes can e.g. concern the (non-)use of potentially burdensome life-prolonging interventions such as hospitalisations or cardio-pulmonary resuscitation. Patients are encouraged to discuss their preferences and questions they may encounter with their physician. The content of the communication during these meetings will be structured by the use of interview guides.

### Study procedures

For each participating hospital, baseline background data will be collected, such as number of cancer patients attending annually, academic/nonacademic setting, number of beds and palliative care services, and a description of common practices regarding ACP and decision-making at the end-of-life. In addition, background reports for each of the six participating countries will be created summarizing baseline national and local policies related to the provision of palliative care and ACP.

We will carefully translate the Respecting Choices program into the required European languages and adapt its content, in close collaboration with the US developers, to the specific legal, clinical, ethical, and cultural contexts of the participating European countries. To test the intervention and the process for acceptability and efficiency, a feasibility study will be conducted with five patients and potentially their family caregiver in each country. The patients will be offered the ACP program and will subsequently be interviewed. We will also test the questionnaires and have conversations with their healthcare providers.

Extensive training of the ACP facilitators is essential in this project. We will use the well-established structure of the training and implementation of the Respecting Choices program and will adopt a two-step education process. First, one representative per country will be trained in La Crosse, Wisconsin (USA) by the instructors of the Respecting Choices program. Subsequently, the country representative will train the local facilitators, who will be --where possible- selected among the healthcare workers of the hospitals, e.g. nurses. All together about 40 facilitators will be trained in the project.

Patients will be followed until one year after inclusion. During the inclusion period eligible patients in both intervention and control hospitals, will be approached for written informed consent. The information provided in the consent form for the intervention group and the control group will be as similar as possible to avoid selection bias with respect to interest in ACP. However, to minimize contamination, patients will be informed that the project aims at investigating the experiences of patients with different approaches towards medical decision-making in advanced stages of cancer, but no or limited details of the Respecting Choices program will be revealed in the control group. Patients will be given ample time to consider participation and they are free to withdraw from participating in the study without any effect on their care.

Patients in the intervention group will be offered the Respecting Choices program in addition to their usual care. Depending on the health status of the patient and the content of the conversations, a facilitated interview will last 45–60 min on average. We plan to have one or two sessions per patient. The facilitator will assist the patient in documenting preferences, including the assignment of a personal representative. For quality assurance, the interviews will be audio recorded by the facilitator.

By a standardized checklist a proportion of the interviews will be rated for intervention fidelity [16].

Ethical committee procedures have been followed in all countries and institutions involved, and approval has been provided. The names of the main IRB’s are:

- The Netherlands: Medische Ethische Toetsings Commissie (METC) ErasmusMC;
- Belgium: Universitair Ziekenhuis Brussel Commissie Medische Ethiek;
- United Kingdom: NRES Committee North West - Liverpool East;
- Italy: Comitato Etico Area Vasta Centro, Regione Toscana;
- Denmark: De Videnskabsetiske Komiteer for Region Hovedstaden;
- Slovenia: Komisija Republike Slovenije za medicinsko etiko (KME).

Approval was also obtained from the IRB’s of all the remaining institutions.

The trial is registered in the International Standard Randomised Controlled Trial Number (ISRCTN63110516). A Data Steering Monitoring Board (DSMB) will be established.

### Measurements

In ACTION, the following measurements will be performed (see Table 2):

**Table 2:** Patient and bereaved carer endpoints of the project

I. Measured by questionnaire	Measure
*Primary endpoints:*	
- Quality of life	EORTC QLQ-C30 4-item emotional functioning scale [24]
	EORTC emotional functioning short-form based on CAT item bank
- Symptoms	EORTC QLQ-C15-PAL [25]
*Secondary endpoints:*	
- Shared decision-making	APECC [26]
- Patient involvement	Self-constructed questions
- Satisfaction with care	EORTC IN–PATSAT32 [27]
- Coping with illness	COPE [28–30]
- Satisfaction with the intervention	Self-constructed questions
- Socio demographic measures	Self-constructed questions
- Quality of end-of-life care	VOICES-SF [31]^a^
- Bereaved carer wellbeing	HADS [32]; IES [33]^a^
II. Obtained from medical files	
- Survival; date and place of death (if applicable)	
- Completion and content of advance directives; preferences for care; assignment of proxy decision-maker; physician orders	
- Diagnostic procedures and treatments received by the patient, hospitalisations and specialist palliative care input.	
III. Obtained from intervention sessions and qualitative interviews	
Systematic cross-cultural comparison of patient experiences, responses and concerns.	

^a^These endpoints are measured by the bereaved carer questionnaire and not by the patient questionnaire

- Questionnaire study. Patients will be asked to complete a written questionnaire about quality of life, symptoms, the decision-making process, patient activation, coping, and satisfaction with care (and the intervention) at baseline (i.e., the moment of inclusion, before the ACP program is delivered in the intervention group), and at 2.5 and 4.5 months after inclusion. If a patient dies during follow up (i.e., within one year after inclusion), a relative identified by the patient as next of kin will receive a questionnaire to assess the patient’s quality of end-of-life care and the relative’s own wellbeing.
- Medical file study. Data on patients’ survival will be collected, as well as preferences as documented and care as received to assess whether patients’ preferred care was congruent with received care. Data on care as received will also be used in the cost-effectiveness analysis. These medical files will be studied one year post-inclusion with a checklist.
- Study of recorded ACP sessions. Data will be obtained from audio recorded facilitated interview sessions. Compliance with the intervention will be systematically evaluated with a predefined checklist.

### Data management

Our data collection tool GemsTracker will be used to safely store data of all participating patients across hospitals and countries. GemsTracker enables restricted access to selected parts of its content. Legislation in the participating countries for research on humans, not involving medical products, will be taken into account [17–22].

### Power calculation, sample size and feasibility of recruitment

With at least 11 intervention and 11 control hospitals each recruiting 34 lung cancer patients and 34 colorectal cancer patients (of which 25 in each tumour type group are expected to remain in the study until at least month 2.5), this multicentre cluster-randomised clinical trial has an overall power of 90 % to identify a minimum difference between intervention and control groups of half a standard deviation on the emotional functioning scale of the QLQ-C30 scale, assuming an intra-class correlation (ICC) of 0.1. On country level, these numbers give a power of 50 % to show such a difference (assuming an ICC of 0.05).

The main outcomes are measured at 2.5 months post-inclusion. Although included patients have an average life expectancy of at least 3 months, we expect that a number of them will die within 2.5 months after inclusion. Based on Dutch colorectal and lung cancer survival statistics [23], we conservatively assume that this will be the case for 15 % of included patients. Furthermore, we anticipate that around 10 % of included patients may drop out of the study for other reasons, resulting in a total attrition rate of 25 %. Based on this attrition rate and an estimated willingness of patients to participate of 33 %, the total number of eligible patients per hospital per cancer type needs to be 101 in a 2-year period, which is feasible in the participating hospitals.

### Analyses

Analyses of the primary and secondary endpoints will be performed following the intention-to-treat principle. Descriptive statistics will be used to summarize characteristics of countries, hospitals and patients. Patient characteristics (age, gender, socio-economic class, educational level) will be compared at baseline between the intervention and control group. A multilevel modelling approach will be used to examine differences in the endpoints between the intervention and control groups, taking account of clustering effects at both hospital and country-level. All statistical tests will be two-sided and considered significant if *p* < 0.05. Repeated-measures analyses of variance will be conducted to assess the development of endpoints over time.

Subgroup analysis will be conducted by means of formal interaction tests for intervention and those variables which are more likely to influence the effect of the intervention itself: gender, age class (<65, 65–74, 75+), educational status, and country.

Those conducting the data analysis will be blinded as to whether the patient was included in the intervention group or in the control group.

### Qualitative study

A complementary qualitative study will be carried out in at least 3 of the 6 countries, to qualitatively explore the lived experience of engagement with the Respecting Choices intervention from the perspectives of patients, their Personal Representatives, healthcare providers and Respecting Choices facilitators. The patient and Personal Representative will undertake a facilitated advance care planning (ACP) conversation following the Respecting Choices program. Within two weeks of completing the ACP program they will be invited to take part in a baseline qualitative interview about their experiences. A follow up interview will occur 10–14 weeks after the initial intervention. At this second interview the patient will be asked whether he or she has discussed the Respecting Choices intervention with anyone from the healthcare team and for consent to contact this person. If the patient dies before the second interview, the Personal Representative will be contacted and invited for a qualitative interview. This will not be arranged until a minimum of six weeks after the patient’s death. Healthcare professionals identified by the patient as being closely involved in the care will be invited to participate in a single face to face, Skype or telephone interview. Respecting Choices facilitators will be invited to participate in a single focus group discussion. In each of the participating countries, the qualitative study will involve between 6–10 cases including a patient and where appropriate a Personal Representative and healthcare professionals. All interviews and focus groups will be recorded and transcribed verbatim. Data will be thematically analysed using a pre-defined coding framework which will be developed through an iterative process of discussion and consensus among the research team.

### Cost-effectiveness study

The economic evaluation will be performed from a healthcare perspective, for a period of one year post-inclusion per patient. Data on total in-hospital medical care will be obtained from medical files, using a standardized and piloted data extraction form. Medical costs will be calculated by multiplying the volumes of healthcare use with the corresponding unit prices. Unit prices will be calculated for all six countries separately. Costs for inpatient days in hospital will be estimated as real, basic costs per day using detailed administrative information. For other cost prices we will use charges. The unit price of the ACP intervention will be determined with the micro-costing method, which is based on a detailed assessment of all resources used. To compare the relative costs and outcomes of ACP versus ‘care as usual’ we will calculate the Incremental Cost Effectiveness Ratio (ICER); the average additional costs of ACP divided by the average change in emotional functioning measured with the EORTC-QLQ-C30 emotional functioning subscale (4 items). A sensitivity analysis will be performed to assess the stability of the results to changes in costs and effectiveness parameters (EORTC QLQ-C15-PAL quality of life subscale), and differences in healthcare systems between the European countries.

### Dissemination

We have set up an Advisory Board of future international policy users of the project results. The role of the Advisory Board will be to provide a critical perspective throughout the life of the project. The project results will be disseminated through publications in scientific journals and conferences. To disseminate the knowledge to all stakeholders we will use the project website (www.action-acp.eu). A link of ACTION to the websites of the consortium and Advisory Board members will be featured.

## Discussion

This project aims to study the effects of the Respecting Choices program on quality of life and symptoms of patients with advanced lung or colorectal cancer. This study has several strengths. First, studies about Advance Care Planning have mainly been performed with older nursing home patients. Transferring the concept of ACP from care of the elderly to patients with advanced cancer, who on average are younger and remain competent for a larger part of their disease trajectory, is a highly relevant next step in an era of increasing focus on patient centered healthcare and shared decision-making. Second, a randomised controlled trial design will enable us to draw conclusions about the causal relations between ACP and the outcomes under study. The clustered design of this project prevents contamination between the control and intervention group. Third, the unique combination of quantitative and qualitative methods in this project will result in profound insights into the underlying working mechanisms of ACP.

In ACTION, we expect to encounter some challenges and possible limitations. First, patients may decline participation for different reasons. They may feel overwhelmed by the topics raised in the ACP intervention sessions and may not (yet) feel prepared to talk about these issues. We will use a patient-centered approach to facilitate study participation. Patients will receive information about the project through their treating specialist. Since patients may refuse because they do not want to engage in ACP conversations, non-response bias cannot be ruled out. Also selection bias cannot be ruled out, e.g. in intervention hospitals’ where including physicians may be more likely to ask patients who they think are more ‘open’ to ACP to participate in the study. If such ‘gatekeeping’ comes into play, the effect of the intervention may be overestimated. However, our approach to systematically assess all lung and colorectal cancer patients for eligibility, and subsequently invite all who are eligible to participate in the study may reduce this risk. Attrition is another potential limitation to this project. Attrition may occur because the condition of the patient might worsen such that further participation becomes impossible, or patients might die during follow-up. We try to limit attrition by adding the inclusion criterion of a minimal anticipated life-expectancy of three months and to measure our main outcome measure at 2.5 months. Third, the international character of this project might be a challenge, as a balance needs to be found between on the one hand testing a uniform intervention in the six countries, that on the other hand is tailored to the specific cultural, ethical and legal context of each country. Fourth, the extent to which actual care will be reflected in medical files can be questioned. Potentially, not all treatments that patients receive will be documented in the hospital medical files.

## Conclusion

Advanced cancer typically involves multiple symptoms and seriously affects patients’ quality of life. Focusing care at patients’ preferences and open and respectful communication are important values in end-of-life care, yet these have been found to be a challenge for healthcare professionals as well as for patients and relatives. Little is known about the outcomes of formal ACP, the effects of formal ACP on medical care and medical decision-making, costs and cost-effectiveness of formal ACP and country-specific factors that might influence ACP. Our project will fill these gaps in knowledge, based on an international multicenter cluster-randomised clinical trial to test the outcomes and effects of a formal ACP program, which is enriched by a qualitative study and a cost-effectiveness study.

Contact: www.action-acp.eu

## Abbreviations

ACP: Advance Care Planning
ACTION: Advance Care Planning; an Innovative Palliative Care Intervention to Improve Quality of Life in Cancer patients – a Multi-Centre Cluster Randomised Clinical Trial
DSMB: Data Steering Monitoring Board
EORTC: European Organisation for Research and Treatment of Cancer
ICC: Intra-Class Correlation
ICER: Incremental Cost Effectiveness Ratio
QLQ: Quality of Life Questionnaire

## References

[1] Ferlay J, Shin HR, Bray F, Forman D, Mathers C, Parkin DM (2010). Estimates of worldwide burden of cancer in 2008: GLOBOCAN 2008. Int J Cancer.

[2] Higginson IJ, Costantini M (2008). Dying with cancer, living well with advanced cancer. Eur J Cancer.

[3] Ferlay J, Autier P, Boniol M, Heanue M, Colombet M, Boyle P (2007). Estimates of the cancer incidence and mortality in Europe in 2006. Ann Oncol.

[4] Brown LF, Kroenke K, Theobald DE, Wu J, Tu W (2010). The association of depression and anxiety with health-related quality of life in cancer patients with depression and/or pain. Psychooncology.

[5] Teno JM, Fisher ES, Hamel MB, Coppola K, Dawson NV (2002). Medical care inconsistent with patients’ treatment goals: association with 1-year Medicare resource use and survival. J Am Geriatr Soc.

[6] Borreani C, Brunelli C, Bianchi E, Piva L, Moro C, Miccinesi G (2012). Talking about end-of-life preferences with advanced cancer patients: factors influencing feasibility. J Pain Symptom Manage.

[7] Visser M, Deliens L, Houttekier D (2014). Physician-related barriers to communication and patient-and family-centred decision-making towards the end of life in intensive care: a systematic review. Crit Care.

[8] Shalowitz DI, Garrett-Mayer E, Wendler D (2006). The accuracy of surrogate decision makers: a systematic review. Arch Intern Med.

[9] Seymour J, Horne G. Advance care planning for the end of life: An overview. In: Thomas K, Lobo B, editors. Advance care planning in end of life care. Oxford: Oxford University Press; 2011. 16–27.

[10] National End of Life Care Programme. Advance Care Planning: A Guide for Health and Social Care Staff, 2nd ed. Leicester. 2008. http://www.ncpc.org.uk/sites/default/files/AdvanceCarePlanning.pdf. Accessed 9 July 2014.

[11] Brinkman-Stoppelenburg A, Rietjens JA, van der Heide A (2014). The effects of advance care planning on end-of-life care: a systematic review. Palliat Med.

[12] Detering KM, Hancock AD, Reade MC, Silvester W (2010). The impact of advance care planning on end of life care in elderly patients: randomised controlled trial. BMJ.

[13] Molloy DW, Guyatt GH, Russo R, Goeree R, O’Brien BJ, Bedard M (2000). Systematic implementation of an advance directive program in nursing homes: a randomized controlled trial. JAMA.

[14] Morrison RS, Chichin E, Carter J, Burack O, Lantz M, Meier DE (2005). The effect of a social work intervention to enhance advance care planning documentation in the nursing home. J Am Geriatr Soc.

[15] Respecting Choices® Advance Care Planning. http://respectingchoices.org Accessed 05-08-2015.

[16] Gearing RE, El-Bassel N, Ghesquiere A, Baldwin S, Gillies J, Ngeow E (2011). Major ingredients of fidelity: A review and scientific guide to improving quality of intervention research implementation. Clin Psychol Rev.

[17] United Nations Educational, Scientific and Cultural Organization. Universal Declaration on Bioethics and Human Rights. 2005. http://unesdoc.unesco.org/images/0014/001461/146180e.pdf. Accessed 9 July 2014.

[18] Council of Europe. Bioethics Division, Oviedo: Convention on Human Rights and Biomedicine (Convention of Oviedo), Articles 15–18. 1997. http://conventions.coe.int/Treaty/en/Treaties/Html/164.htm. Accessed 9 July 2014.

[19] Council of Europe. Bioethics Division. Strasbourg: Additional Protocol to the Convention on Human Rights and Biomedicine, concerning Biomedical Research. 2005. http://conventions.coe.int/Treaty/en/Treaties/Html/195.htm. Accessed 9 July 2014.

[20] World Medical A (2013). World Medical Association Declaration of Helsinki: ethical principles for medical research involving human subjects. JAMA.

[21] Council for International Organizations of Medical Sciences. 1991. http://www.psi.uba.ar/academica/carrerasdegrado/psicologia/sitios_catedras/obligatorias/723_etica2/material/normativas/cioms_epidemiological_studies.pdf. Accessed 9 July 2014.

[22] Council for International Organizations of Medical Sciences. 2002. http://www.recerca.uab.es/ceeah/docs/CIOMS.pdf. Accessed 9 July 2014.

[23] Integraal Kankercentrum Nederland. Cijfers over kanker. 2011. http://cijfersoverkanker.nl/. Accessed 9 July 2014.

[24] Aaronson NK, Ahmedzai S, Bergman B, Bullinger M, Cull A, Duez NJ (1993). The European Organization for Research and Treatment of Cancer QLQ-C30: a quality-of-life instrument for use in international clinical trials in oncology. J Natl Cancer Inst.

[25] Groenvold M, Petersen MA, Aaronson NK, Arraras JI, Blazeby JM, Bottomley A (2006). The development of the EORTC QLQ-C15-PAL: a shortened questionnaire for cancer patients in palliative care. Eur J Cancer.

[26] Arora NK, Weaver KE, Clayman ML, Oakley-Girvan I, Potosky AL (2009). Physicians’ decision-making style and psychosocial outcomes among cancer survivors. Patient Educ Couns.

[27] Bredart A, Bottomley A, Blazeby JM, Conroy T, Coens C, D’Haese S (2005). An international prospective study of the EORTC cancer in-patient satisfaction with care measure (EORTC IN-PATSAT32). Eur J Cancer.

[28] Carver CS (1997). You want to measure coping but your protocol’s too long: consider the brief COPE. Int J Behav Med.

[29] Carver CS, Scheier MF, Weintraub JK (1989). Assessing coping strategies: a theoretically based approach. J Pers Soc Psychol.

[30] Stanton AL, Kirk SB, Cameron CL, Danoff-Burg S (2000). Coping through emotional approach: scale construction and validation. J Pers Soc Psychol.

[31] Hunt KJ, Shlomo N, Richardson A, Addington-Hall J (2011). VOICES redesign and testing to inform a national end of life care survey.

[32] Snaith RP (2003). The hospital anxiety and depression scale. Health Qual Life Outcomes.

[33] Zigmond AS, Snaith RP (1983). The hospital anxiety and depression scale. Acta Psychiatr Scand.

